# Enrichment of cancer-predisposing germline variants in adult and pediatric patients with acute lymphoblastic leukemia

**DOI:** 10.1038/s41598-022-14364-x

**Published:** 2022-06-23

**Authors:** Suvi P. M. Douglas, Atte K. Lahtinen, Jessica R. Koski, Lilli Leimi, Mikko A. I. Keränen, Minna Koskenvuo, Caroline A. Heckman, Kirsi Jahnukainen, Esa Pitkänen, Ulla Wartiovaara-Kautto, Outi Kilpivaara

**Affiliations:** 1 Applied Tumor Genomics Research Program, Faculty of Medicine, University of Helsinki, Helsinki, Finland; 2 Department of Medical and Clinical Genetics, Medicum, Faculty of Medicine, University of Helsinki, Helsinki, Finland; 3 Children’s Hospital, and Pediatric Research Center, University of Helsinki and Helsinki University Hospital, Helsinki, Finland; 4 Department of Hematology, Helsinki University Hospital Comprehensive Cancer Center, University of Helsinki, Helsinki, Finland; 5 Hematology Research Unit Helsinki, University of Helsinki, Helsinki, Finland; 6 Division of Hematology-Oncology and Stem Cell Transplantation, New Children’s Hospital, University of Helsinki and Helsinki University Hospital, Helsinki, Finland; 7 Institute for Molecular Medicine Finland (FIMM), University of Helsinki, Helsinki, Finland; 8 Department of Women’s and Children’s Health, Karolinska Institutet and University Hospital, Solna, Sweden; 9 HUSLAB Laboratory of Genetics, HUS Diagnostic Center, Helsinki University Hospital, Helsinki, Finland

**Keywords:** Acute lymphocytic leukaemia, Medical genomics, Personalized medicine, Medical genetics

## Abstract

Despite recent progress in acute lymphoblastic leukemia (ALL) therapies, a significant subset of adult and pediatric ALL patients has a dismal prognosis. Better understanding of leukemogenesis and recognition of germline genetic changes may provide new tools for treating patients. Given that hematopoietic stem cell transplantation, often from a family member, is a major form of treatment in ALL, acknowledging the possibility of hereditary predisposition is of special importance. Reports of comprehensive germline analyses performed in adult ALL patients are scarce. Aiming at fulfilling this gap of knowledge, we investigated variants in 93 genes predisposing to hematologic malignancies and 70 other cancer-predisposing genes from exome data obtained from 61 adult and 87 pediatric ALL patients. Our results show that pathogenic (P) or likely pathogenic (LP) germline variants in genes associated with predisposition to ALL or other cancers are prevalent in ALL patients: 8% of adults and 11% of children. Comparison of P/LP germline variants in patients to population-matched controls (gnomAD Finns) revealed a 2.6-fold enrichment in ALL cases (CI 95% 1.5–4.2, *p* = 0.00071). Acknowledging inherited factors is crucial, especially when considering hematopoietic stem cell transplantation and planning post-therapy follow-up. Harmful germline variants may also predispose patients to excessive toxicity potentially compromising the outcome. We propose integrating germline genetics into precise ALL patient care and providing families genetic counseling.

## Introduction

Acute lymphoblastic leukemia (ALL) affects individuals of all ages and is the most common pediatric cancer. Although ALL is aggressive and quickly progressing, it usually reacts well to initial treatment^1^. However, ten to twenty percent of pediatric and 30–60% of adult patients with ALL relapse and high-risk disease remains a challenge^2,3^. Intensification of ALL therapy by performing allogeneic hematopoietic stem cell transplantation (HSCT) from a family member or registry donor is currently the first option of treatment. Also new treatment modalities, such as targeted antibodies and CAR-T cell therapy are entering the clinics^2,4^.

The studies in solid malignancies have paved the way for integration of germline genetic information into the care of cancer patients. The identification of inherited genetic variants may affect the choice of treatment in multiple ways and enable early intervention. However, in hematological malignancies, the recognition of these factors has only recently taken root in clinical practice; Constitutional genetic changes are now acknowledged in acute myeloid leukemias (AML) and included in the newest WHO classification of AML^5,6^. Regarding ALL, data and practices on germline alterations predisposing to the disease are based mostly on pediatric, family, and genome-wide association studies (GWAS), and only one report presenting exome analysis on adult ALL patients (n = 28) has been published^7^.

Two types of ALL susceptibility have been reported: rare germline mutations with high-penetrance predisposition, such as *IKZF1*^8^, *PAX5*^9,10^, and *ETV6*^11,12^, and common variants with low-penetrance predisposition identified in GWASs^13,14^ (e.g. *ARID5B*^15^, *GATA3*^16^, and *ERG*^17^)*.* Furthermore, in epidemiological studies including also non-pediatric ALL patients, a higher risk of leukemia has been observed in family members, which speaks for shared genetic factors behind the disease^18,19^.

Here, we have evaluated the extent and distribution of pathogenic (P) and likely pathogenic (LP) germline variation in known leukemia and solid malignancy predisposing genes by performing a comprehensive exome analysis in both adult and pediatric ALL patients.

## Methods

### Patients

We examined whole exome sequencing (WES) data on freshly collected or biobanked samples of adult (n = 61, aged 16–70) and pediatric (n = 87, aged 0–16) patients with ALL (Finnish Hematological Registry and Biobank – FHRB, and clinical repositories). Adults were diagnosed in the Helsinki University Hospital during the years 2008–2019. WES of adult patients was performed on skin samples except for one remission blood sample. The pediatric samples were collected from ALL patients treated with allogeneic HSCT according to the treatment protocols^1,20,21^ in the Helsinki University Hospital pediatric unit during 1998–2019. WES in these cases was performed on blood samples taken at the time of HLA typing for HSCT. Specimen preparation and WES are described in detail in Supplementary methods. We also had access to all patients’ clinical information and results of laboratory tests performed during treatment. Patient characteristics are summarized in Table 1 and detailed information on patients is listed in Supplementary Table S1.

**Table 1:** Summary of patient characteristics and source of DNA.

	Adults	Children
**Number of patients**	61	87
Male	40 (66%)	59 (68%)
Female	21 (34%)	28 (32%)
Age at dg (y)	16–70	0–16
Mean age at dg (y)	38	7
**ALL type**		
B-ALL	36 (59%)	66 (76%)
T-ALL	17 (28%)	11 (13%)
Ph-ALL	8 (13%)	10 (11%)
Source of DNA	Skin^a^	Blood^b^

Dg, diagnosis; y, years; B-ALL, Ph-negative B-ALL; Ph-ALL, Ph-positive B-ALL.

^a^One adult patient’s DNA was extracted from blood, but after recovering from ALL.

^b^See methods for determining the germline/somatic origin of variants.

### Ethics declarations

This study has been approved by Helsinki University Hospital ethics review committee (adults #206/13/03/03/2016 and #303/13/03/01/2011; pediatric patients HUS/114/2018, HUS/284/2019 and V/3235/2019). All samples and data were derived either after written informed consent (living individuals), or authorization by the ethics committee (deceased patients). All methods were conducted in accordance with the relevant guidelines and regulations.

### Analyses of the germline variants

We constructed a list of 93 genes (Supplementary Table S2) known to predispose to ALL or other hematological malignancies based on literature^22,23^. Additionally, a list of 70 genes reported with germline predisposition to cancer (Supplementary Table S3) was compiled based on COSMIC cancer gene census^24^ (version 94, Tier 1, https://cancer.sanger.ac.uk/census) and NHGRI Clinical Genomics Database (https://research.nhgri.nih.gov/CGD/, accessed 05/03/2021). Variant analysis (workflow presented in Supplementary Figure S1) was done using BasePlayer^25^, a tool for analyzing and visualizing next-generation sequencing data. Only non-synonymous and splice site variants in protein-coding transcripts (except for *TERC*, which is a ncRNA, *ANKRD26* UTR variants and *GATA2* synonymous variants) with a minor allele frequency (MAF) of < 0.01 in both the gnomAD non-cancer whole database and gnomAD non-cancer Finns (version 2.1)^26^ were considered. Variants filtered in gnomAD due to poor quality were discarded. A 1000 genomes mappability pilot mask track^27^ and quality measures—genotype quality (GQ) > 20, QUAL > 20, site total coverage > 6 reads, and allelic fraction ≥ 20%—were used to filter out poor-quality variants. All variants were also visually confirmed using BasePlayer: at least two unique reads in both directions with the alternate allele were required and dubious variants in repetitive sequence regions were discarded. For low-coverage variants, also the somatic exome (blood or bone marrow) of the same patient was used to verify the variant, if available. The median coverage was 64 × and 232 × for adult and pediatric samples, respectively.

**Figure 1:**
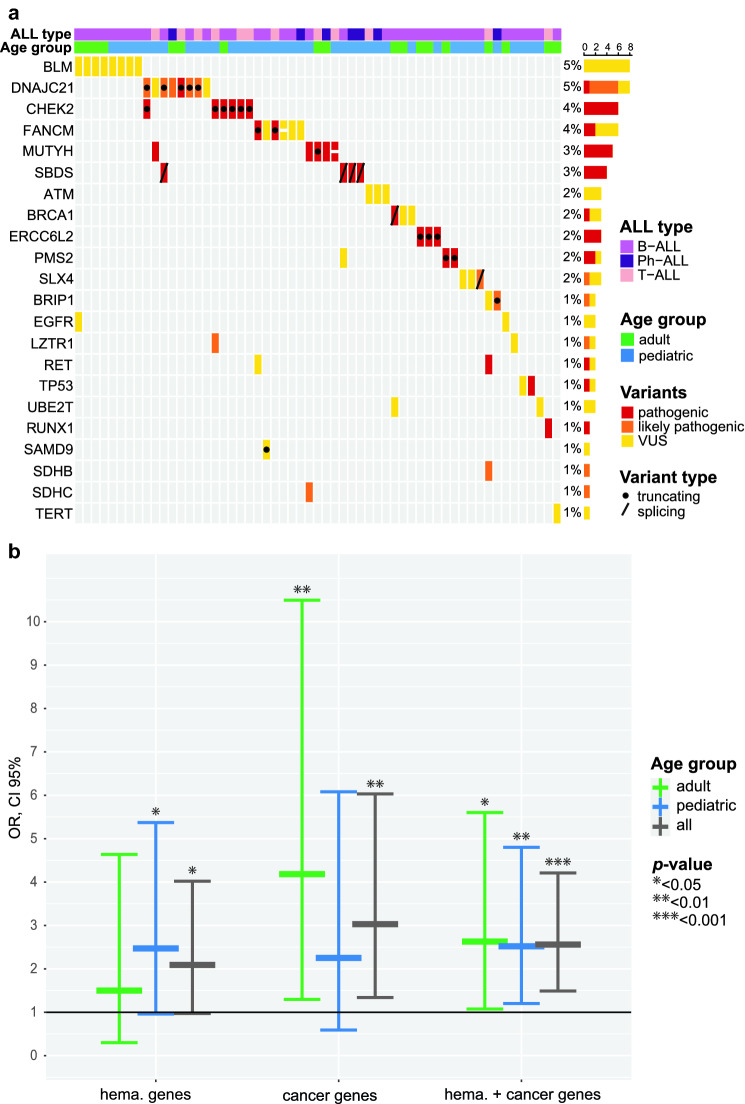
(**a**) The distribution of all pathogenic and likely pathogenic variants and variants of uncertain significance (VUS) in the ALL patients. The frequency and number of patients harboring a variant in each gene are shown on the right-hand panel. B-ALL, Ph-negative B-ALL; Ph-ALL, Ph-positive B-ALL. (**b**) Enrichment of pathogenic variants in ALL patients compared to healthy controls (gnomAD Finns). Variants in genes with known predisposition to hematological malignancies (n = 93) and other known cancer-predisposition genes (n = 70) are shown separately and together for both age groups and overall. OR, odds ratio; CI, confidence interval.

The classification of the germline variants (benign, likely benign, variant of uncertain significance (VUS), likely pathogenic (LP), or pathogenic (P)), according to the American College of Medical Genetics (ACMG) guidelines^28^, was done first with Intervar^29^ (version 2.0.2). Thereafter, to confirm the findings, the P/LP/VUS variants were classified with Varsome^30^ (ACMG version 9.5.0, https://varsome.com/) since differences in interpretation exist due to various databases and prediction algorithms used. Our own conclusive judgment of pathogenicity for the variants (used throughout the manuscript) was based on careful consideration of all the rules and evidence from InterVar, Varsome and literature. Predictions by both tools are reported in the Table 2 and Supplementary Table S4. The oncoplot in Fig. 1a was made using ComplexHeatmap package (version 2.10.0)^31^ and the boxplot in Fig. 1b was made using ggplot2 (version 3.3.5, https://ggplot2.tidyverse.org)^32^ in R (version 4.1.2, https://www.r-project.org/)^33^. As knowledge on the actionable germline variants is still evolving, we also report variants of uncertain significance, if P/LP variants were identified in the study patients in the respective genes by either of the used prediction tools. The VUS were not included in the calculations of the reported frequencies of (P/LP) variants. Exceptions to the above-mentioned interpretation rules were made with *BLM*, due to identification of surprisingly high number (7) of the same VUS. Also, a VUS in *TERT* is reported because it has been shown to correlate with short telomeres^34^ (Supplementary Table S4).

**Table 2:** Pathogenic/likely pathogenic variants in autosomal dominant genes or compound heterozygous variants in recessive genes.

Patient	Age	Type	Gene	Inheritance	Germline disease association for gene	ACMG Intervar	ACMG Varsome	Conclusion of pathogenicity	Variant HGVS	gnomAD all MAF	gnomAD Finns MAF
2181	41	B-ALL	BRCA1	AR;AD	Fanconi anemia; Breast cancer	P	P	P	c.4097-2A > G (splicing)	0	0
2150	32	B-ALL	CHEK2	AD	Li-Fraumeni syndrome 2	VUS	P	P	c.1100del (p.Thr367MetfsTer15)	0.00205	0.00874
2149	43	B-ALL	PMS2	AR;AD	CMMRD; HNPCC	P	P	P	c.765C > A (p.Tyr255Ter)	0	0
2307*	53	T-ALL	RET	AD	Multiple endocrine neoplasia	VUS	P	P	c.2410G > A (p.Val804Met)	0.00011	0
2167	54	T-ALL	RUNX1	AD	Familial platelet disorder with predisposition to AML	LP	P	P	c.611G > A (p.Arg204Gln)	0	0
2307*	53	T-ALL	SDHB	AD	Familial paraganglioma-pheochromocytoma	VUS	LP	LP	c.177G > C (p.Gln59His)	0.000004	0
P2220^†^	8	Ph-ALL	BRIP1	AR;AD	FA; Breast cancer	VUS	P	LP	c.3440dup (p.Asn1147LysfsTer2)	0.00009	0.00068
P2206	15	B-ALL	CHEK2	AD	Li-Fraumeni syndrome 2	VUS	P	P	c.1100del (p.Thr367MetfsTer15)	0.00205	0.00874
P2209^†^	15	B-ALL	CHEK2	AD	Li-Fraumeni syndrome 2	VUS	P	P	c.1100del (p.Thr367MetfsTer15)	0.00205	0.00874
P2249*	2	T-ALL	CHEK2	AD	Li-Fraumeni syndrome 2	VUS	P	P	c.1100del (p.Thr367MetfsTer15)	0.00205	0.00874
P2257^†^	14	T-ALL	CHEK2	AD	Li-Fraumeni syndrome 2	VUS	P	P	c.1100del (p.Thr367MetfsTer15)	0.00205	0.00874
P2431	9	T-ALL	CHEK2	AD	Li-Fraumeni syndrome 2	VUS	P	P	c.1100del (p.Thr367MetfsTer15)	0.00205	0.00874
P2249*	2	T-ALL	LZTR1	AR/AD;AD	Noonan syndrome, Schwannomatosis	P	P	LP	c.2407-1G > A (splicing)	0.00002	0.00018
P2216*	8	T-ALL	MUTYH	AR	Familial adenomatous polyposis	LP	P	P	c.1187G > A (p.Gly396Asp)	0.00295	0.0022
P2216*	8	T-ALL	MUTYH	AR	Familial adenomatous polyposis	LP	P	P	c.536A > G (p.Tyr179Cys)	0.00154	0.00153
P2255	0	B-ALL	PMS2	AR;AD	CMMRD; HNPCC	P	P	P	c.325dup (p.Glu109GlyfsTer30)	0.00002	0
P2261	0	B-ALL	SDHC	AD	Familial paraganglioma-pheochromocytoma	LP	P	LP	c.380A > G (p.His127Arg)	0	0
P2224	2	B-ALL	TP53	AD	Li-Fraumeni syndrome	LP	P	P	c.733G > A (p.Gly245Ser)	0	0
						Total P/LP 10 variants,9 patients^a^	Total P/LP 20 variants, 17 patients^a^	Total P/LP 18 variants, 15 patients			

ACMG, American College of Medical Genetics; AD, autosomal dominant; AML, acute myeloid leukemia; AR, autosomal recessive; B-ALL, Ph-negative B-ALL; Ph-ALL, Ph-positive B-ALL; CMMRD, constitutional mismatch repair deficiency; FA, Fanconi anemia; HNPCC, hereditary non-polyposis colorectal cancer; LP, likely pathogenic; MAF, minor allele frequency; P, pathogenic; VUS, variant of uncertain significance.

*Patients with multiple P/LP variants.

^†^Samples with a high blast percentage.

^a^All variants classified P/LP by Intervar and Varsome not shown here. Pediatric patients are marked with a P.

### Determining the germline/somatic origin of the pediatric patients’ variants

The exome sequenced blood samples from pediatric patients were obtained at different phases of ALL with a varying degree of blastemia. Germline variants’ variant allele fraction (VAF) should be close to 0.5 for heterozygous variants and 1.0 for homozygous variants. We classified the variants as somatic or germline manually by considering the blast percentage, VAF and karyotype in each sample. To support the interpretation of the origin of the variant, we also verified if the variant was reported in the gnomAD database, ClinVar^35^ (germline variants) (https://www.ncbi.nlm.nih.gov/clinvar/), or in cBioPortal^36,37^ (https://www.cbioportal.org/) (somatic variants) (Supplementary Table S5). Variants that have been reported in normal population and never as somatic, are likely germline, and vice versa. This is even more pronounced in Finland, where certain germline variants are enriched and therefore these variants are even more likely to be of germline origin (Supplementary Table S5). Only variants which were evidently of germline origin were included in the analyses. If the judgement was uncertain, the variant was not interpreted as of germline origin. All variants in patients with ≥ 50% blasts are reported in Supplementary Table S5, along with justification of germline origin of the variant.

### Enrichment of pathogenic or likely pathogenic variants

To define the potential enrichment of rare (MAF < 0.01) P/LP variants in the studied genes, we compared the frequency of all rare P/LP variants in these gene sets with respect to ACMG classification in adult and pediatric ALL patients and in gnomAD non-cancer Finns^26^ v.2.1 (n = 21,632 alleles). We also compared the frequency of all P/LP variants in each studied gene separately (with at least one P/LP variant) in patients and gnomAD non-cancer Finns. The differences were evaluated using 2-sided Fisher exact tests. Benjamini–Hochberg procedure was used to adjust the *p*-values for multiple testing. In genes with multiple truncating variants that were determined as VUS, we compared the frequencies of truncating variants only. As an exception for other analyses in this study, we used Intervar as the only prediction tool here since it is open source and feasible to be integrated into pipeline to classify large amounts of variants.

## Results

### Frequency and description of the identified germline variants in ALL patients

Overall, by using stringent classification criteria 11/61 (18%) of adults and 21/87 (24%) of children had heterozygous P/LP variants in the genes analyzed (Fig. 1a, Supplementary Table S4). If we excluded the heterozygous variants in genes where the acknowledged mode of heritance is autosomal recessive, the respective numbers were 5/61 (8%) in adult and 10/87 (11%) in pediatric patients (Table 2). Most of these were previously reported P variants with a known cancer risk—in 5/61 (8%) adults and 8/87 (9%) pediatric patients. All the identified P/LP/VUS are demonstrated in the oncoplot (Fig. 1a), and the classification criteria in the Supplementary Table S4.

We discovered one P variant in high-risk (not lineage-restricted) leukemia predisposition genes: *RUNX1* p.Arg204Gln in an adult patient. A truncating variant in *SAMD9* p.Ser844ValfsTer10 was found in a pediatric patient. We classified it as a VUS since functional information and segregation analysis on this variant were not available, and most of the pathogenic variants in *SAMD9* are missense variants. However, a few rare loss-of-function variants in *SAMD9* have also been identified in myelodysplastic syndrome patients^38,39^. Most malignancies caused by germline *RUNX1* and *SAMD9* variants are myeloid, but also predisposition to lymphoid malignancies has been reported^40,41^. Regarding genes causing syndromes with a risk of ALL, we found two P/LP variants in Li-Fraumeni and Noonan syndrome -causing genes: one P variant in *TP53* (p.Gly245Ser) and one in *LZTR1* (c.2407-1G > A) (Fig. 1a, Table 2).

We also detected six heterozygous truncating P/LP variants and two VUS missense variants in *DNAJC21*, in which autosomal recessively inherited mutations have been reported to cause Shwachman-Diamond-like disease^42^.

Seven patients had P/LP variants in colorectal cancer risk -associated genes *MUTYH* and *PMS2*. Four of them had a single P variant in *MUTYH –* p.Arg19Ter (n = 1) and hotspot variants p.Gly396Asp (n = 2), and p.Tyr179Cys (n = 1); while one pediatric patient was compound heterozygous for the hotspot variants p.Gly396Asp and p.Tyr179Cys. In *PMS2* we identified two pathogenic heterozygous variants (p.Tyr255Ter and p.Glu109GlyfsTer30) in one adult and one pediatric patient, respectively. There was no known family history of colorectal cancer in these patients.

We also identified heterozygous P/LP variants in genes predisposing to other solid tumors: *BRCA1* (n = 1), *BRIP1* (n = 1) and *CHEK2* (n = 6), that are breast cancer –predisposing genes, *RET* (n = 1), which predisposes to multiple endocrine neoplasia, and *SDHB* (n = 1) and *SDHC* (n = 1) that are known to predispose familial paraganglioma- pheocromocytoma (Table 2, Fig. 1a).

### The comparison of the frequency of harmful germline variants with population-matched controls

We compared the frequency of P/LP germline predisposition variants in ALL patients to population-matched (Finns) GnomAD non-cancer control data. We found a 2.6–fold (CI 95% 1.5–4.2, *p* = 0.00071) enrichment of variants in the adult and pediatric patient sets combined (Fig. 1b, Supplementary Table S6). The enrichment of variants was pronounced in traditional cancer predisposition genes in adults, and in hematological malignancy genes in children, 4.2- and 2.5-fold, respectively (Fig. 1b, Supplementary Table S6).

Statistically, the most significant differences in the frequency of P/LP germline variants between patients and population-matched non-cancer controls were detected in *PMS2, DNAJC21* (truncating variants only), and *MUTYH (*OR ranging from 4.1 to 48.9) (Supplementary Table S7). P/LP variants in colorectal cancer risk—associated genes were enriched in our ALL patients compared to non-cancer Finns (*PMS2* OR 48.91, 95% CI 4.08–434.55; *p* = 0.0018, *MUTYH* OR 5.12, 95% CI 1.82–11.74, *p* = 0.0016). Despite the recessive inheritance of *DNAJC21*, enrichment of truncating heterozygous variants was seen in the study patients compared to population-matched (gnomAD non-cancer Finns) controls (OR 4.07, 95% CI 1.28–9.95, *p* = 0.00976). All significant (*p* < 0.05) results, except *CHEK2,* survived correction for multiple testing (FDR < 10%) (Supplementary Table S7).

## Discussion

In this study we present data on germline alterations in pediatric and adult ALL patients. Previous reports on this matter and especially in adult cases are scarce. In the analyses, we took advantage of having a large population-specific reference dataset in Finland to assess the variant enrichment in the patients compared to population-matched non-cancer controls. This is, to our knowledge, the largest assessment of predisposition variants in adult ALL patients performed to date, which is important given the inferior survival of adult ALL patients. Altogether, we identified likely harmful germline variants in 8% of adult and 15% of pediatric ALL patients. These rates are parallel to results attained from studies on myeloid malignancies^6,43^. A previous study on childhood cancers found germline predisposition in childhood ALL in ~ 4% of the patients^44^. In our pediatric series the frequency was more than three-fold. However, as Zhang et al. discuss, their results may be underestimates. Furthermore, our study included only children with high-risk ALL who had undergone allogeneic HSCT, in distinction to Zhang et al*.* report. A study on Korean ALL patients (65 pediatric, 28 adult cases) found only 1.1% carrying pathogenic variants in ALL-predisposing syndrome genes and one multi-exon deletion^7^. Interestingly, both pathogenic variants were discovered in adult patients (> 16yrs) (2/28). Evidently, additional studies and larger material are needed, especially on adult ALL, to further define the role of germline predisposition in ALL.

When focusing on the variants we discovered, we didn’t find any variants in genes predisposing solely to ALL. Nevertheless, we identified a rare harmful variant in RUNX1, first recognized in AML. This *RUNX1* variant seen in patient 2167 has been reported in a family with AML and T-ALL^40^. Also, our patient suffered from T-cell ALL, the rarer lineage but typical for germline *RUNX1* mutation-linked lymphoblastic leukemia^40,41^. Interestingly, the patient’s family history of cancer did not suggest a high-penetrance germline mutation.

In children, two rare harmful variants were identified in genes causing syndromes with ALL predisposition: one in *TP53* and one in *LZTR1*. Germline *TP53* mutations cause Li-Fraumeni syndrome, which is characterized by a high risk for malignancies, including ALL, in particular hypodiploid ALL^45^. In our patient with the germline *TP53* mutation, ALL (not hypodiploid) was the first presentation of Li-Fraumeni syndrome (at the age 2). An increased risk for leukemias in general, but also in ALL, has been described in Noonan syndrome patients with different germline mutations causing the disease^46,47^. Patients with Noonan syndrome may have some characteristic features, such as cardiac abnormalities and facial features, but there were no indications of these in our patients. Noonan syndrome -causing *LZTR1* variants have been reported in the literature both in homozygous and heterozygous forms, depending on the variant^48,49^. The LP *LZTR1* splice site variant (c.2407-1G > A) identified in our study has not been reported before, but a variant in the adjacent base (c.2407-2A > G) has been reported in Noonan syndrome patients (homozygous and one heterozygote in ClinVar) and in schwannomatosis patients^49,50^. This suggests that the variant is damaging.

Homozygous or compound heterozygous mutations in *DNAJC21* have been reported to cause Shwachman-Diamond -like hematological phenotype^42^. We identified several heterozygous variants in our data, which is statistically more than observed in the control set. The implication of these *DNAJC21* variants in leukemogenesis needs to be validated in other patient sets and functional experiments before further interpretation.

In addition to the genes known to predispose to hematological malignancies, patients harbored several interesting variants in genes primarily associated with an increased risk for solid tumors (*MUTYH, PMS2,* and *CHEK2*). Also, for some genes the mode of inheritance is not unequivocal. MUTYH is involved in base excision repair and biallelic germline mutations in it cause MUTYH-associated polyposis. Although *MUTYH* is considered an AR predisposition gene, several studies also suggest a modestly increased cancer risk for heterozygous mutation carriers^51–53^. Specifically, the hotspot variant p.Gly396Asp seen in our patients, has been suggested to result in increased risk for some cancers, such as small intestinal neuroendocrine tumors and a modest increase in breast cancer risk as monoallelic^52,53^. Germline *PMS2* mutations cause hereditary nonpolyposis colorectal cancer (AD) and constitutional mismatch repair deficiency syndrome (AR).The *CHEK2* variant c.1100delC is not a very rare variant in Finnish population, but it is a known pathogenic variant with a modestly increased breast cancer risk^54^. Also, predisposition to other solid malignancies has been reported^55^, given the universal role of CHEK2 in DNA repair.

In Finland we have the advantage of having a large population-specific reference dataset (gnomAD Finns^26^ v.2.1, n = 21,632 alleles). This enables more precise assessment of variant enrichment in the patients. In general, we found an enrichment of P/LP variants in cancer-predisposing genes in the ALL patients compared to population-matched control data. However, our results are transferable, as the prevalence of ALL in Finland is comparable with other developed countries, according to the published registry data^56^.

Since we took into account only P/LP variants (and excluded VUS) and the gene lists comprised only of currently known predisposition genes, this result presents the minimum number of rare germline predisposition variants. We identified P/LP germline variants also in adult ALL patients, and therefore, germline predisposition should not be ignored in ALL in any age groups. Our patients are, however, more high-risk than average, but it is not known if the predisposition to ALL is related to the severity of the disease. To our knowledge our exome data set in adult ALL patients is unique and even for pediatric patients only few estimates on the frequency of germline predisposition to ALL have been reported.

The difference in the frequency between patients and controls was more pronounced in hematological genes in children and in cancer genes in adults. The explanation for this may be explained by the fact that the list of hematological malignancy genes consists of high-penetrance ALL predisposition factors found originally in pediatric patient sets and leukemia families. Furthermore, those harboring high-penetrance predisposition mutations for ALL are more likely to have the disease in childhood. However, more studies are needed to estimate more precisely the gene/variant -specific risk. The clustering of the solid malignancy predisposition gene variants in the adult patient set, instead, may indicate their contribution to leukemogenesis later in life. Identifying carriers of general cancer predisposition gene defects—often involved in DNA repair—should not be ignored. This is especially reflected in the setting of allogeneic HSCT given to 20–50% of adult and 5–10% of pediatric ALL patients. The procedure comprises of multiple steps where germline defects may interfere donor selection from family-members, intensity of conditioning therapy, and need for individualized surveillance plan regarding risks for second or secondary malignancies or late toxicities.

This study was not aimed at finding causal connection between variants and ALL, but to analyze the frequency of P/LP variants in genes possibly contributing to ALL pathogenesis and their enrichment in ALL patients. Identification of harmful rare germline variants may, however, contribute to the selection of optimal therapy (HSCT from siblings/donor registry, possible new treatments). It may also affect the surveillance plan of the patients regarding the risk of secondary or second cancers, and genetic counseling^57^.

Certainly, some ALL-predisposing genes remain unveiled. The rarity of ALL in adults makes it more difficult to find new adult-specific genes with ALL predisposition without exceptional families. We found some P/LP variants in genes with high penetrance for hematological malignancies among our study patients despite not focusing on families with multiple ALL cases. Family history is only indicative, but not definitive of germline predisposition and mutations can also arise de novo*.*

## Conclusions

A proportion of ALL patients of all ages, carries P/LP germline variants in cancer-predisposing genes even without clear indications for germline testing (such as personal or family history of leukemia or other cancer). In contrast to pediatric ALL patients, the possibility of inherited predisposition in adults has been oversighted in the clinical practice. Acknowledging inherited factors is crucial, especially when considering HSCT donors and planning post-therapy follow-up. Harmful germline variants may also predispose patients to excessive toxicity potentially compromising the outcome. Furthermore, recognition of the role of germline factors in leukemogenesis may also provide new tools for therapy development. Analogous to AML, we propose integrating germline genetics into precise ALL patient care and providing genetic counseling to affected families.

## Supplementary Information

Supplementary Information 1.

Supplementary Information 2.

## Data Availability

The datasets generated and/or analyzed during the current study are not publicly available due to privacy and ethical restrictions but are available from the corresponding author upon reasonable request.
