# Supplementary material for: Enrichment of cancer-predisposing germline variants in adult and pediatric patients with acute lymphoblastic leukemia
Source: Sci Rep. 2022 Jun 23;12:10670. doi: 10.1038/s41598-022-14364-x (PMC9225984; doi:10.1038/s41598-022-14364-x)
Supplement: Supplementary file 2 — Supplementary Information 2. [file 41598_2022_14364_MOESM2_ESM.pdf]

Enrichment of cancer-predisposing germline variants in adult and pediatric patients with acute lymphoblastic leukemia

Suvi P.M. Douglas, Atte K. Lahtinen, Jessica R. Koski, Lilli Leimi, Mikko A.I. Keränen, Minna Koskenvuo, Caroline A. Heckman, Kirsi Jahnukainen, Esa Pitkänen, Ulla Wartiovaara-Kautto, and Outi Kilpivaara

SUPPLEMENTARY DATA

SUPPLEMENTARY METHODS 2

Exome sequencing of adult samples 2

Exome sequencing of pediatric samples 2

SUPPLEMENTARY FIGURES 3

Supplementary Figure S1. The workflow of the analysis of germline variants in ALL patients. 3

SUPPLEMENTARY TABLES 4

Supplementary Table S1. Patient characteristics and identified variants. (Separate file.) 4

Supplementary Table S2. Genes with risk to hematological malignancy. 4

Supplementary Table S3. Genes associated with cancer predisposition 6

Supplementary Table S4. Identified P/LP/VUS variants. 8

Supplementary Table S5. Variants in patients with >50% blasts. 11

Supplementary Table S6. Enrichment of P/LP variants. 12

Supplementary Table S7. Enrichment of P/LP variants in each gene. 13

SUPPLEMENTARY REFERENCES 14

SUPPLEMENTARY METHODS

Exome sequencing of adult samples

DNA was extracted from skin samples using DNeasy Blood and Tissue kit according to the manufacturer's protocol (Qiagen, Hilden, Germany). For one patient, only remission blood sample was available, and the DNA was extracted with NucleoSpin DNA Blood XL kit (Macherey-Nagel, Düren, Germany). WES of the adults' samples was conducted at the Institute for Molecular Medicine Finland (FIMM, Helsinki, Finland) or Biomedicum Functional Genomics Unit (FuGU, Helsinki, Finland) as described previously[1] using Nimblegen SeqCap EZ exome v2.0 (Roche, Basel, Switzerland), The SeqCap EZ MedExome (Roche, Basel, Switzerland) or Agilent Clinical Research Exome (Agilent, Santa Clara, CA, USA) kits.

Exome sequencing of pediatric samples

Exome sequencing and sample preparation of pediatric patients was performed at Blueprint Genetics (Helsinki, Finland). Total genomic DNA was extracted from the samples using a spin column method. The DNA samples were randomly fragmented using non-contact, isothermal sonochemistry processing and purified with SPRI beads. DNA fragments were then end-repaired and sequencing adapters were ligated to both ends of the resulting fragments. Prepared DNA-Adapter libraries were size-selected with SPRI beads to ensure optimal template size and then amplified by ligation-mediated PCR. The amplified sequencing library was purified using SPRI beads and a hybridization-capture method was used for enrichment of whole exome and select non-coding regions (xGen Exome Research Panel with custom-designed capture probes (Integrated DNA Technologies, Coralville, Iowa, USA). The quality of the completed sequencing library was controlled by ensuring the correct template size and quantity and to eliminate the presence of leftover primer-dimers. Captured libraries were sequenced using the Illumina sequencing system with paired-end sequencing (2x150 bp). Raw sequence reads were quality controlled and clean reads were mapped to the human reference genome (GRCh37/hg19) with Burrows-Wheeler Aligner (BWA-MEM) software[2]. Duplicate read marking, local realignment around indels, base quality score recalibration and variant calling were performed using GATK[3].

SUPPLEMENTARY FIGURES

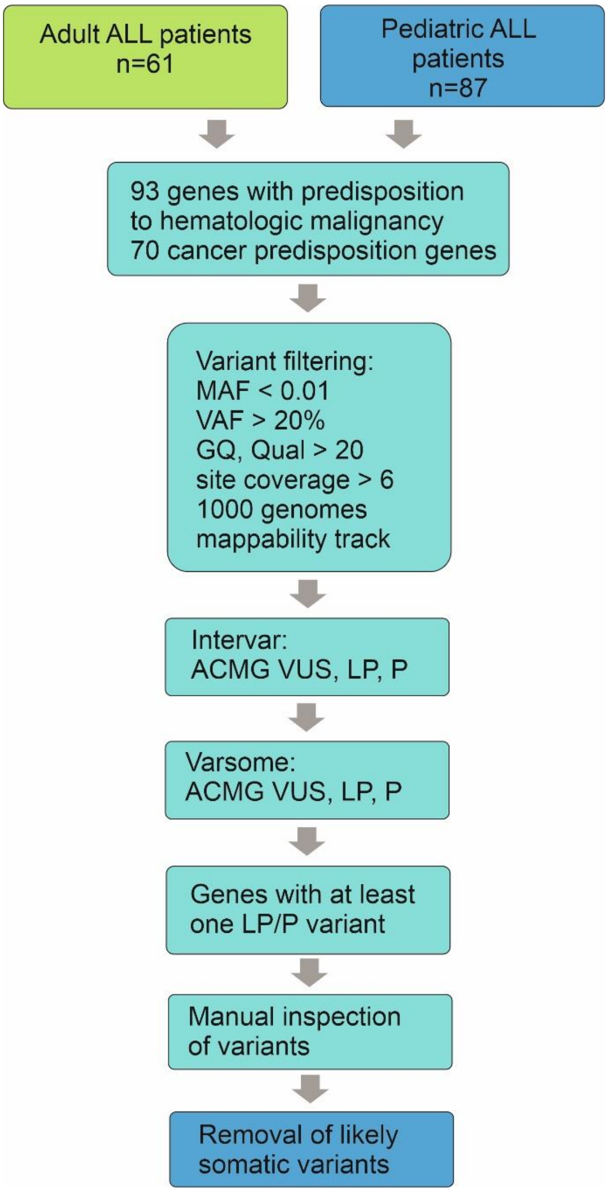

Supplementary Figure S1. The workflow of the analysis of germline variants in ALL patients. MAF, minor allele frequency; VAF, variant allele frequency; GQ, genotype quality; ACMG, American College of Medical Genetics classification; VUS, variant of unknown significance; LP, likely pathogenic; P, pathogenic.

SUPPLEMENTARY TABLES

Supplementary Table S1. Patient characteristics and identified variants. (Separate file.) ACMG, American College of Medical Genetics; AD, autosomal dominant; ALL, acute lymphoid leukemia; AML, acute myeloid leukemia; AR, autosomal recessive; BMF, bone marrow failure; DKC, dyskeratosis congenita; dx, diagnosis; F, female; GI, gastrointestinal; HNPCC, Hereditary nonpolyposis colorectal cancer; LBL, lymphoblastic lymphoma; LP, likely pathogenic; M, male; MAF, minor allele frequency; MDS, myelodysplastic syndrome; MMRCS, mismatch repair cancer syndrome, NOS, not otherwise specified; N/A, not available; P, pathogenic; SDS, Shwachman-Diamond syndrome; VUS, variant of uncertain significance. [a]ALL type: B-ALL, Ph-neg B-ALL; Ph-ALL, Ph+ B-ALL [b]Analyzed fusion genes: *SIL-TAL1, KMT2A-EPS15, KMT2A-MLLT11, TCF3-PBX1, NPM1-MLF1, RUNX-MECOM, KMT2A-AFF1, ETV6-PDGFRB, NPM1-RARA, DEK-NUP214, KMT2A-AFDN, RUNX-RUNX1T1, SET-NUP214, KMT2A-MLLT3, ETV6-ABL1, BCR-ABL1, KMT2A-MLLT10, KMT2A-MLLT6, ZBTB16-RARA, KMT2A-ELL, KMT2A-MLLT1, ETV6-RUNX1, ETV6-MN1, PML-RARA, CBFB-MYH11, FUS-ERG, TCF3-HLF, KMT2A-FOXO4* [c]Non-Finnish origin (Patient 2162 originally from Egypt, 2166 from Iraq and patient 2307 originally from Somalia)

Supplementary Table S2. Genes with risk to hematological malignancy. AD, autosomal dominant; ALL, acute lymphoblastic leukemia, AML, acute myeloid leukemia; AR, autosomal recessive; CVID, common variable immunodeficiency; MDS, myelodysplastic syndrome; XL, X-linked.

Gene	Condition	Inheritance	References
ACD	Dyskeratosis congenita	AD/AR	[4,5]
ANKRD26	Thrombocytopenia 2, myeloid malignancies	AD	[6]
ATM	Breast cancer susceptibility; ataxia-telangiectasia	AD;AR	[7–9]
BLM	Bloom syndrome	AR	[10,11]
BRCA1	Familial breast-ovarian cancer, pancreatic cancer susceptibility; Fanconi anemia	AD;AR	[12–14]
BRCA2	Familial breast-ovarian cancer, pancreatic cancer susceptibility; Fanconi anemia	AD;AR	[12–15]
BRIP1	Breast cancer susceptibility; Fanconi anemia	AD;AR	[13,15]
CEBPA	Familial acute myeloid leukemia	AD	[16]
DDX41	Familial myelo/lymphoproliferative neoplasm susceptibility	AD	[17]
DKC1	Dyskeratosis congenita	XL	[4,18]
DNAJC21	Bone marrow failure syndrome 3	AR	[19]
EFL1	Shwachman-Diamond syndrome	AR	[19]
ERCC4	Fanconi anemia, xeroderma pigmentosum	AR	[13]
ERCC6L2	Bone marrow failure syndrome 2, acute myeloid leukemia	AR	[20,21]
ETV6	Thrombocytopenia 5, ALL predisposition	AD	[22–26]
FANCA	Fanconi anemia	AR	[13,15]
FANCB	Fanconi anemia	XL	[13]
FANCC	Fanconi anemia	AR	[13,15]
FANCD2	Fanconi anemia	AR	[13,15]
FANCE	Fanconi anemia	AR	[13,15]
FANCF	Fanconi anemia	AR	[13,15]
FANCG	Fanconi anemia	AR	[12,13]
FANCI	Fanconi anemia	AR	[12,13]
FANCL	Fanconi anemia	AR	[12,13]
FANCM	Premature ovarian failure 15	AR	[12,13]
GAB2	Putative ALL predisposition	AD	[27]
GATA1	Thrombocytopenia with beta-thalassemia, Diamond-Blackfan anemia	XL	[28]
GATA2	Immunodeficiency 21, Emberger syndrome, MDS/AML susceptibility	AD	[29]
IKZF1	CVID, ALL predisposition	AD	[30–34]
LIG4	LIG4 syndrome	AR	[35]
MAD2L2	Fanconi anemia	AR	[13,36]
MBD4	Myeloid malignancies	AR	[37]

MLH1	Lynch syndrome; CMMRD	AD;AR	38
MSH2	Lynch syndrome; CMMRD	AD;AR	38
MSH6	Lynch syndrome; CMMRD	AD;AR	38
MYH9	Macrothrombocytopenia and granulocyte inclusions with or without nephritis or sensorineural hearing loss	AD	39
NBN	Breast cancer susceptibility; Nijmegen breakage syndrome	AD;AR	9,40
NF1	Neurofibromatosis type I	AD	41,42
NHP2	Dyskeratosis congenita	AR	4
NOP10	Dyskeratosis congenita	AR	4
NPM1	Dyskeratosis congenita	AD	43
PALB2	Breast and pancreatic cancer susceptibility; Fanconi anemia	AR	13
PARN	Pulmonary fibrosis and/or bone marrow failure, telomere-related 4; Dyskeratosis congenita	AD;AR	4
PAX5	ALL susceptibility	AD	44,45
PMS2	Lynch syndrome; CMMRD	AD;AR	38
POT1	Familial chronic lymphocytic leukemia	AD	5
PTPN11	Noonan syndrome	AD	9,46,47
PTPRJ	Inherited thrombocytopenia	AR	48
RAD51	Fanconi anemia	AD	13
RAD51C	Breast-ovarian cancer susceptibility; Fanconi anemia	AD; AR	13
RECQL4	Baller-Gerold syndrome, RAPADILINO syndrome, Rothmund-Thomson syndrome	AR	49
RFWD3	Fanconi anemia	AR	50
RPL11	Diamond-Blackfan anemia	AD	28
RPL15	Diamond-Blackfan anemia	AD	28
RPL18	Diamond-Blackfan anemia	AD	51
RPL26	Diamond-Blackfan anemia	AD	28
RPL27	Diamond-Blackfan anemia	AD	28
RPL31	Diamond-Blackfan anemia	AD	28
RPL35	Diamond-Blackfan anemia	AD	51
RPL35A	Diamond-Blackfan anemia	AD	28
RPL5	Diamond-Blackfan anemia	AD	28
RPL9	Diamond-Blackfan anemia	AD	28
RPS10	Diamond-Blackfan anemia	AD	28
RPS15	Diamond-Blackfan anemia	AD	28
RPS15A	Diamond-Blackfan anemia	AD	52
RPS17	Diamond-Blackfan anemia	AD	28
RPS19	Diamond-Blackfan anemia	AD	53
RPS24	Diamond-Blackfan anemia	AD	28
RPS26	Diamond-Blackfan anemia	AD	28
RPS27	Diamond-Blackfan anemia	AD	28
RPS27A	Diamond-Blackfan anemia	AD	28
RPS28	Diamond-Blackfan anemia	AD	28
RPS29	Diamond-Blackfan anemia	AD	28
RPS7	Diamond-Blackfan anemia	AD	28
RTEL1	Dyskeratosis congenita	AD/AR	4
RUNX1	Familial platelet disorder with associated myeloid malignancy	AD	6,54,55
SAMD9	Mirage syndrome, Monosomy 7 myelodysplasia and leukemia syndrome	AD	56,57
SAMD9L	Ataxia-pancytopenia syndrome, Monosomy 7 myelodysplasia and leukemia syndrome	AD	57,58
SBDS	Shwachman-Diamond syndrome; (Aplastic anemia?)	AR; AD?	59
SH2B3	ALL and MPN susceptibility	AR	60
SLX4	Fanconi anemia	AR	13
SRP72	Bone marrow failure syndrome 1	AD	61

TERC	Dyskeratosis congenita	AD	[4]
TERT	Dyskeratosis congenita	AD/AR	[4]
TET2	Myeloid malignancies and lymphoma susceptibility; Immunodeficiency 75	AD;AR	[62]
TINF2	Dyskeratosis congenita	AD	[4]
TP53	Li-Fraumeni syndrome, ALL and myeloid malignancies	AD	[63]
TSR2	Diamond-Blackfan anemia	XL	[28]
TYK2	putative ALL predisposition; Immunodeficiency 35	AD;AR	[64]
UBE2T	Fanconi anemia	AR	[13]
WAS	Wiskott-Aldrich syndrome	XL	[65]
WRAP53	Dyskeratosis congenita	AR	[4]
XRCC2	Fanconi anemia	AR	[13]

Supplementary Table S3. Genes associated with cancer predisposition. Seventy other genes that are associated with germline cancer predisposition. AD, autosomal dominant: AR, autosomal recessive; XL, X-linked.

Gene	Inheritance	Condition
ALK	AD	familial neuroblastoma
APC	AD	adenomatous polyposis coli, Turcot syndrome
AR	XL	prostate cancer predisposition
ATR	AD;AR	familial cutaneous telangiectasia and cancer syndrome; Seckel syndrome
AXIN2	AD	oligodontia-colorectal cancer syndrome
BAP1	AD	BAP1 tumor predisposition syndrome
BARD1	AD	breast cancer susceptibility
BMPR1A	AD	juvenile polyposis
BUB1B	AD;AR	mosaic variegated aneuploidy; premature chromatid separation trait
CDC73	AD	hyperparathyroidism-jaw tumour syndrome, parathyroid carcinoma
CDH1	AD	familial gastric carcinoma
CDK4	AD	familial malignant melanoma
CDKN1B	AD	multiple endocrine neoplasia
CDKN2A	AD	familial malignant melanoma
CHEK2	AD	familial breast cancer
CXCR4	AD	WHIM syndrome
CYLD	AD	familial cylindromatosis, trichoepithelioma, Brooke-Spiegler syndrome
DDB2	AR	xeroderma pigmentosum
DICER1	AD	DICER1 syndrome
EGFR	AR	lung cancer susceptibility
ERBB4	AD	amyotrophic lateral sclerosis
ERCC2	AR	xeroderma pigmentosum
ERCC3	AR	xeroderma pigmentosum
ERCC5	AR	xeroderma pigmentosum
EXT1	AD	multiple exostoses type 1, chondrosarcoma
EXT2	AD	multiple exostoses type 2
FH	AD	hereditary leiomyomatosis and renal cell cancer
FLCN	AD	Birt-Hogg-Dube syndrome
GPC3	XL	Simpson-Golabi-Behmel syndrome
HNF1A	AD	renal cell carcinoma, familial hepatic adenoma
HRAS	AD	Costello syndrome

KIT	AD	familial gastrointestinal stromal tumour
LZTR1	AD/AR	schwannomatosis, Noonan syndrome
MAX	AD	pheochromocytoma
MEN1	AD	multiple endocrine neoplasia type 1
MPL	AD;AR	familial essential thrombocythemia
MUTYH	AR	familial adenomatous polyposis
NF2	AD	neurofibromatosis type 2
PDGFRA	AD	familial gastrointestinal stromal tumour
PHOX2B	AD	familial neuroblastoma
POLD1	AD	colorectal cancer susceptibility
POLE	AD;AR	colorectal cancer susceptibility; FILS syndrome, IMAGE-I syndrome
PRF1	AR	familial hemophagocytic lymphohistiocytosis, lymphoma, aplastic anemia
PRKAR1A	AD	Carney complex
PTCH1	AD	basal cell nevus syndrome
PTEN	AD	Cowden syndrome
RB1	AD	familial retinoblastoma
RET	AD;AR	multiple endocrine neoplasia
SDHA	AD;AR	paragangliomas; gastrointestinal stromal tumors, Leigh syndrome
SDHAF2	AD	familial paraganglioma
SDHB	AD	familial paraganglioma
SDHC	AD	familial paraganglioma
SDHD	AD	familial paraganglioma
SETBP1	AD	Schinzel-Giedion syndrome
SMAD4	AD	juvenile polyposis
SMARCB1	AD	rhabdoid tumor predisposition syndrome
SMARCE1	AD	famial meningioma
SPOP	AD	prostate cancer
STAT3	AD	paediatric large granular lymphocytic leukaemia
STK11	AD	Peutz-Jeghers syndrome
SUFU	AD	medulloblastoma predisposition
TGFBR2	AD	hereditary nonpolyposis colorectal cancer
TMEM127	AD	pheochromocytoma
TSC1	AD	tuberous sclerosis 1
TSC2	AD	tuberous sclerosis 2
VHL	AD	Von Hippel-Lindau syndrome
WRN	AR	Werner syndrome
WT1	AD	Denys-Drash syndrome, Frasier syndrome, familial Wilms tumour
XPA	AR	xeroderma pigmentosum
XPC	AR	xeroderma pigmentosum

Supplementary Table S4. Identified P/LP/VUS variants. Pathogenic/Likely pathogenic variants and variants of uncertain significance in genes with a risk for hematologic malignancy or cancer in general. Pediatric patients are marked with P and include blast percentage in blood sample for interpreting the origin of variants. ACMG, American college of medical genetics verdict; AD, autosomal dominant; AML, acute myeloid leukemia; AR, autosomal recessive; CMMRD, constitutional mismatch repair deficiency; DKC, dyskeratosis congenita; FA, Fanconi anemia; HNPCC, hereditary non polyposis colorectal cancer; MAF, minor allele frequency; SDS, Shwachman-Diamond syndrome. [a]Varsome rule strengths: VS, very strong; S, strong; Mo, moderate; Su, supporting.

Patient	Gene	Inheritance	Predisposition to	Variant	Position (GRCh37)	ACMG classification Intervar	ACMG classification Varsome[a]	Conclusion of pathogenicity	VAF	Blasts (blood)	MAF gnomAD all	MAF gnomAD Finns
**P2238**	*ATM*	AD;AR	Ataxia-Telangiectasia; Breast cancer	NM_000051.4:c.6539G>T (p.Gly2180Val)	11:108192114	VUS (PM1,PM2,PP3,BP1)	VUS (PM1,PM2 - Mo; BP4 - Su)	VUS	0.51	0	0.00001	0.00014
**P2244**	*ATM*	AD;AR	Ataxia-Telangiectasia; Breast cancer	NM_000051.4:c.7390T>C (p.Cys2464Arg)	11:108201023	VUS (PM1,PM2,PP3,BP1)	VUS (PM1,PM2 - Mo; BP4 - Su)	VUS	0.5	0.04	0.00042	0.00005
**P2251**	*ATM*	AD;AR	Ataxia-Telangiectasia; Breast cancer	NM_000051.4:c.6067G>A (p.Gly2023Arg)	11:108186610	VUS (PM1,PM2,PP3,BP1)	LP (PM1,PM2 - Mo; PP3,PP5 - Su)	VUS	0.47	0	0.00142	0.00083
**2143**	*BLM**	AR	Bloom syndrome	NM_000057.4:c.2362C>A (p.Leu788Ile)	15:91312417	VUS (PM1,PM2,PP3,BP1)	VUS (PM1,PM2 - Mo; PP3 - Su)	VUS	0.54	-	0.00093	0.00587
**2146**	*BLM**	AR	Bloom syndrome	NM_000057.4:c.2362C>A (p.Leu788Ile)	15:91312417	VUS (PM1,PM2,PP3,BP1)	VUS (PM1,PM2 - Mo; PP3 - Su)	VUS	0.54	-	0.00093	0.00587
**2175**	*BLM**	AR	Bloom syndrome	NM_000057.4:c.2362C>A (p.Leu788Ile)	15:91312417	VUS (PM1,PM2,PP3,BP1)	VUS (PM1,PM2 - Mo; PP3 - Su)	VUS	0.54	-	0.00093	0.00587
**2188**	*BLM**	AR	Bloom syndrome	NM_000057.4:c.2119C>T (p.Pro707Ser)	15:91308570	VUS (PM1,PM2,PP3,BP1)	VUS (PM1,PM2 - Mo; BP4 - Su)	VUS	0.49	-	0.00163	0.00037
**P2235**	*BLM**	AR	Bloom syndrome	NM_000057.4:c.2362C>A (p.Leu788Ile)	15:91312417	VUS (PM1,PM2,PP3,BP1)	VUS (PM1,PM2 - Mo; PP3 - Su)	VUS	0.41	0.71	0.00093	0.00587
**P2430**	*BLM**	AR	Bloom syndrome	NM_000057.4:c.2362C>A (p.Leu788Ile)	15:91312417	VUS (PM1,PM2,PP3,BP1)	VUS (PM1,PM2 - Mo; PP3 - Su)	VUS	0.48	0	0.00093	0.00587
**P2434**	*BLM**	AR	Bloom syndrome	NM_000057.4:c.2362C>A (p.Leu788Ile)	15:91312417	VUS (PM1,PM2,PP3,BP1)	VUS (PM1,PM2 - Mo; PP3 - Su)	VUS	0.49	0	0.00093	0.00587
**P2447**	*BLM**	AR	Bloom syndrome	NM_000057.4:c.2362C>A (p.Leu788Ile)	15:91312417	VUS (PM1,PM2,PP3,BP1)	VUS (PM1,PM2 - Mo; PP3 - Su)	VUS	0.55	0	0.00093	0.00587
**2171**	*BRCA1*	AR;AD	FA; Breast cancer	NM_007300.4:c.5261A>T (p.Asp1754Val)	17:41209148	VUS (PM1,PP3,BP1)	VUS (PM1,PM2 - Mo; PP3 - Su)	VUS	0.45	-	0.00007	0.00069
**2181**	*BRCA1*	AR;AD	FA; Breast cancer	NM_007300.4:c.4097-2A>G splicing	17:41243051	P (PVS1,PM2,PP3,PP5)	P (PVS1 - VS; PM2 - Mo; PP3 - Su; PP5 - VS)	P	0.58	-	0	0
**P2440**	*BRCA1*	AR;AD	FA; Breast cancer	NM_007300.4:c.4798C>T (p.Pro1600Ser)	17:41223196	VUS (PM2,BP1)	VUS (PM2 - Mo; BP4 - Su)	VUS	0.55	0	0.00001	0.00009
**2307**	*BRIP1*	AR;AD	FA; Breast cancer	NM_032043.3:c.2594G>A (p.Arg865Gln)	17:59763508	VUS (PM1,PM2,PP3,BP1)	VUS (PM2,PM5 - Mo; PP3 - Su)	VUS	0.48	-	0.00001	0
**P2220**	*BRIP1*	AR;AD	FA; Breast cancer	NM_032043.3:c.3440dup (p.Asn1147LysfsTer2)	17:59760966	VUS (PM2)	P (PVS1 - VS; PM2 - Mo; PP3 - Su)	LP	0.48	0.97	0.00009	0.00068
**2150**	*CHEK2*	AD	Li-Fraumeni syndrome 2	NM_007194.4:c.1100del (p.Thr367MetfsTer15)	22:29091856	VUS (PVS1,BS1)	P (PVS1 - VS; PM2 - Mo; PP3 - Su; PP5 - VS)	P	0.43	-	0.00205	0.00874
**P2206**	*CHEK2*	AD	Li-Fraumeni syndrome 2	NM_007194.4:c.1100del (p.Thr367MetfsTer15)	22:29091856	VUS (PVS1,BS1)	P (PVS1 - VS; PM2 - Mo; PP3 - Su; PP5 - VS)	P	0.5	0	0.00205	0.00874
**P2209**	*CHEK2*	AD	Li-Fraumeni syndrome 2	NM_007194.4:c.1100del (p.Thr367MetfsTer15)	22:29091856	VUS (PVS1,BS1)	P (PVS1 - VS; PM2 - Mo; PP3 - Su; PP5 - VS)	P	0.47	0.78	0.00205	0.00874
**P2249**	*CHEK2*	AD	Li-Fraumeni syndrome 2	NM_007194.4:c.1100del (p.Thr367MetfsTer15)	22:29091856	VUS (PVS1,BS1)	P (PVS1 - VS; PM2 - Mo; PP3 - Su; PP5 - VS)	P	0.9	0.45	0.00205	0.00874
**P2257**	*CHEK2*	AD	Li-Fraumeni syndrome 2	NM_007194.4:c.1100del (p.Thr367MetfsTer15)	22:29091856	VUS (PVS1,BS1)	P (PVS1 - VS; PM2 - Mo; PP3 - Su; PP5 - VS)	P	0.42	0.87	0.00205	0.00874
**P2431**	*CHEK2*	AD	Li-Fraumeni syndrome 2	NM_007194.4:c.1100del (p.Thr367MetfsTer15)	22:29091856	VUS (PVS1,BS1)	P (PVS1 - VS; PM2 - Mo; PP3 - Su; PP5 - VS)	P	0.48	0	0.00205	0.00874
**2121**	*DNAJC21*	AR	SDS	NM_001012339.3:c.1594_*1del4 (stop loss)	5:34954814	VUS (PM4)	VUS (PM2,PM4 - Mo; PP3 - Su)	LP	0.51	-	0.00006	0.00033
**2164**	*DNAJC21*	AR	SDS	NM_001012339.3:c.1368del (p.Lys456AsnfsTer10)	5:34954038	VUS (PVS1)	P (PVS1 - VS; PM2 - Mo; PP3 - Su)	P	0.46	-	0.00026	0.00014
**P2203**	*DNAJC21*	AR	SDS	NM_194283.4:c.1240G>T (p.Gly414Ter)	5:34949702	VUS (PVS1)	LP (PVS1 - VS; PM2 - Mo; BP4 - Su)	LP	0.41	0	0.00041	0.00355

**P2206**	*DNAJC21*	AR	SDS	NM_194283.4:c.1240G>T (p.Gly414Ter)	5:34949702	VUS (PVS1)	LP (PVS1 - VS; PM2 - Mo; BP4 - Su)	LP	0.58	0	0.00041	0.00355
**P2212**	*DNAJC21*	AR	SDS	NM_001012339.3:c.370G>C (p.Glu124Gln)	5:34936303	VUS (PP3,BP1)	VUS (PM2 - Mo; PP3,BP1 - Su)	VUS	0.57	0	0.00061	0.00208
**P2234**	*DNAJC21*	AR	SDS	NM_194283.4:c.1240G>T (p.Gly414Ter)	5:34949702	VUS (PVS1)	LP (PVS1 - VS; PM2 - Mo; BP4 - Su)	LP	0.44	0.96	0.00041	0.00355
**P2236**	*DNAJC21*	AR	SDS	NM_194283.4:c.1240G>T (p.Gly414Ter)	5:34949702	VUS (PVS1)	LP (PVS1 - VS; PM2 - Mo; BP4 - Su)	LP	0.48	0.19	0.00041	0.00355
**P2243**	*DNAJC21*	AR	SDS	NM_001012339.3:c.370G>C (p.Glu124Gln)	5:34936303	VUS (PP3,BP1)	VUS (PM2 - Mo; PP3,BP1 - Su)	VUS	0.49	0	0.00061	0.00208
**2158**	*EGFR*	AD	Lung cancer	NM_005228.5:c.1774G>A (p.Val592Ile)	7:55233024	LP (PM1,PM2,PP2,PP3,BS2)	VUS (PM2 - Mo; PP2 - Su)	VUS	0.55	-	0.0003	0.00005
**2175**	*EGFR*	AD	Lung cancer	NM_005228.5:c.844G>A (p.Glu282Lys)	7:55221800	VUS (PM1,PP2,PP3,BS1)	VUS (PM1,PM2 - Mo; PP2,BP4 - Su)	VUS	0.51	-	0.00033	0.00037
**2152**	*ERCC6L2*	AR	BMF, AML	NM_020207.7:c.1424del (p.Ile475ThrfsTer36)	9:98685552	VUS (PVS1,BS1)	P (PVS1 - VS; PM2 - Mo; PP3 - Su; PP5 - VS)	P	0.57	-	0.00048	0.00511
**2165**	*ERCC6L2*	AR	BMF, AML	NM_020207.7:c.1424del (p.Ile475ThrfsTer36)	9:98685552	VUS (PVS1,BS1)	P (PVS1 - VS; PM2 - Mo; PP3 - Su; PP5 - VS)	P	0.46	-	0.00048	0.00511
**P2205**	*ERCC6L2*	AR	BMF, AML	NM_020207.7:c.1424del (p.Ile475ThrfsTer36)	9:98685552	VUS (PVS1,BS1)	P (PVS1 - VS; PM2 - Mo; PP3 - Su; PP5 - VS)	P	0.44	0	0.00048	0.00511
**P2199**	*FANCM*	AR	FA	NM_020937.4:c.5791C>T (p.Arg1931Ter)	14:45667921	P (PVS1,PP3,PP5)	P (PVS1 - VS; PM2 - Mo; PP3 - Su)	P	0.56	0	0.00101	0.00448
**P2208**	*FANCM*	AR	FA	NM_020937.4:c.1555G>A (p.Gly519Ser)	14:45628457	VUS (PM1,PM2,PP3)	VUS (PM2 - Mo; BP4 - Su)	VUS	0.4	0	0	0
**P2218**	*FANCM*	AR	FA	NM_020937.4:c.5101C>T (p.Gln1701Ter)	14:45658326	VUS (PVS1,PP5,BS1)	P (PVS1 - VS; PM2 - Mo; PP3 - Su; PP5 - VS)	P	0.43	0.96	0.00129	0.00823
**P2247**	*FANCM*	AR	FA	NM_020937.4:c.2903A>G (p.Tyr968Cys)	14:45644860	VUS	VUS (PM2 - Mo; BP4 - Su)	VUS	0.48	0	0.00002	0.00005
**P2253**	*FANCM*	AR	FA	NM_020937.4:c.2339A>C (p.Glu780Ala)	14:45644296	VUS (PM1,PP3)	VUS (PM2 - Mo)	VUS	0.76	0.55	0.00003	0
**P2253**	*FANCM*	AR	FA	NM_020937.4:c.2389C>G (p.Pro797Ala)	14:45644346	VUS	VUS (PM2 - Mo; BP4 - Su)	VUS	0.76	0.55	0.00003	0
**P2420**	*FANCM*	AR	FA	NM_020937.4:c.4696A>G (p.Met1566Val)	14:45657007	VUS (PM2)	VUS (PM2 - Mo; BP4 - Su)	VUS	0.58	0	0.00002	0.00005
**P2214**	*LZTR1*	AD/AR	Noonan syndrome	NM_006767.4:c.1711G>A (p.Glu571Lys)	22:21348942	VUS (PM1,PP3)	VUS (PM2 - Mo; PP2 - Su; PP3 - Su)	VUS	0.47	0	0.00007	0.00071
**P2249**	*LZTR1*	AD/AR	Noonan syndrome	NM_006767.4:c.2407-1G>A splicing	22:21351520	P (PVS1,PM2,PP3)	P (PVS1 - VS; PM2 - Mo; PP3 - Su)	LP	0.95	0.45	0.00002	0.00018
**2144**	*MUTYH*	AR	Familial adenomatous polyposis	NM_001128425.2:c.55C>T (p.Arg19Ter)	1:45800165	P (PVS1,PM2,PP3,PP5)	P (PVS1 - VS; PM2 - Mo; PP3 - Su; PP5 - St)	P	0.47	-	0.00001	0
**2177**	*MUTYH*	AR	Familial adenomatous polyposis	NM_001128425.2:c.536A>G (p.Tyr179Cys)	1:45798475	LP (PM1,PM2,PP3,PP5,BP1)	P (PM1,PM2 - Mo; PP2, PP3 - Su; PP5 - VS)	P	0.59	-	0.00154	0.00153
**P2212**	*MUTYH*	AR	Familial adenomatous polyposis	NM_001128425.2:c.1187G>A (p.Gly396Asp)	1:45797228	LP (PM1,PM2,PP3,PP5,BP1)	P (PS3 - St; PM1,PM5 - Mo; PP2 - Su; PP3 - St; PP5 - VS)	P	0.49	0	0.00295	0.0022
**P2216**	*MUTYH*	AR	Familial adenomatous polyposis	NM_001128425.2:c.1187G>A (p.Gly396Asp)	1:45797228	LP (PM1,PM2,PP3,PP5,BP1)	P (PS3 - St; PM1,PM5 - Mo; PP2 - Su; PP3 - St; PP5 - VS)	P	0.46	0	0.00295	0.0022
**P2216**	*MUTYH*	AR	Familial adenomatous polyposis	NM_001128425.2:c.536A>G (p.Tyr179Cys)	1:45798475	LP (PM1,PM2,PP3,PP5,BP1)	P (PM1,PM2 - Mo; PP2, PP3 - Su; PP5 - VS)	P	0.47	0	0.00154	0.00153
**P2261**	*MUTYH*	AR	Familial adenomatous polyposis	NM_001128425.2:c.1187G>A (p.Gly396Asp)	1:45797228	LP (PM1,PM2,PP3,PP5,BP1)	P (PS3 - St; PM1,PM5 - Mo; PP2 - Su; PP3 - St; PP5 - VS)	P	0.46	0	0.00295	0.0022
**2149**	*PMS2*	AR;AD	CMMRD; HNPCC	NM_000535.7:c.765C>A (p.Tyr255Ter)	7:6036995	P (PVS1,PM2,PP5)	P (PVS1 - VS; PM2 - Mo; PP3 - Su; PP5 - St)	P	0.57	-	0	0
**P2213**	*PMS2*	AR;AD	CMMRD; HNPCC	NM_000535.7:c.320G>C (p.Arg107Pro)	7:6043354	VUS (PM1,PM2,PP3,BP1)	VUS (PM2 - Mo; PM5 - Su; PP3 - Su)	VUS	0.4	0.09	0	0
**P2255**	*PMS2*	AR;AD	CMMRD; HNPCC	NM_000535.7:c.325dup (p.Glu109GlyfsTer30)	7:6043348	P (PVS1,PM2,PP5)	P (PVS1 - VS; PM2 - Mo; PP3 - Su)	P	0.56	0	0.00002	0

**2307**	*RET*	AD	Multiple endocrine neoplasia	NM_020975.6:c.2410G>A (p.Val804Met)	10:43614996	VUS (PM1,PM2,PP3)	P (PM1,PM2,PM5 - Mo; PP2,PP3 - Su; PP5 - VS)	P	0.45	-	0.00011	0
**P2199**	*RET*	AD	Multiple endocrine neoplasia	NM_020975.6:c.973G>A (p.Ala325Thr)	10:43601929	VUS (PM2)	VUS (PM2 - Mo; PP2,BP4 - Su)	VUS	0.44	0	0.00002	0
**2167**	*RUNX1*	AD	Familial platelet disorder with predisposition to AML	NM_001754.5:c.611G>A (p.Arg204Gln)	21:36231773	LP (PM1,PM2,PP3,PP5)	P (PM1,PM2,PM5 - Mo; PP2 - Su; PP3 - St; PP5 - VS)	P	0.46	-	0	0
**P2208**	*SAMD9*	AD	Mirage syndrome, AML	NM_001193307.1:c.2530del (p.Ser844ValfsTer10)	7:92732880	VUS (PM2)	P (PVS1 - VS; PM2 - Mo; PP3 - Su)	VUS	0.39	0	0	0
**P2204**	*SBDS*	AR;(AD)	SDS; (aplastic anemia susceptibility)	NM_016038.4:c.258+2T>C splicing	7:66459197	P (PVS1,PP3,PP5,BS1)	P (PVS1 - VS; PM2 - Mo; PP3 - Su; PP5 - VS)	P	0.52	0	0.00397	0.00958
**P2213**	*SBDS*	AR;(AD)	SDS; (aplastic anemia susceptibility)	NM_016038.4:c.258+2T>C splicing	7:66459197	P (PVS1,PP3,PP5,BS1)	P (PVS1 - VS; PM2 - Mo; PP3 - Su; PP5 - VS)	P	0.41	0.09	0.00397	0.00958
**P2223**	*SBDS*	AR;(AD)	SDS; (aplastic anemia susceptibility)	NM_016038.4:c.258+2T>C splicing	7:66459197	P (PVS1,PP3,PP5,BS1)	P (PVS1 - VS; PM2 - Mo; PP3 - Su; PP5 - VS)	P	0.33	0.83	0.00397	0.00958
**P2234**	*SBDS*	AR;(AD)	SDS; (aplastic anemia susceptibility)	NM_016038.4:c.258+2T>C splicing	7:66459197	P (PVS1,PP3,PP5,BS1)	P (PVS1 - VS; PM2 - Mo; PP3 - Su; PP5 - VS)	P	0.45	0.96	0.00397	0.00958
**2307**	*SDHB*	AD	familial paraganglioma - pheochromocytoma	NM_003000.3:c.177G>C (p.Gln59His)	1:17371279	VUS (PM1,PM2)	LP (PM1,PM2 - Mo; PP2,PP3 - Su)	LP	0.54	-	0	0
**P2261**	*SDHC*	AD	familial paraganglioma - pheochromocytoma	NM_003001.5:c.380A>G (p.His127Arg)	1:161326605	LP (PM1,PM2,PP3,PP5)	P (PM1,PM2,PM5 - Mo; PP3 - Su; PP5 - St)	LP	0.47	0	0	0
**P2217**	*SLX4*	AR	FA	NM_032444.4:c.1469T>C (p.Leu490Pro)	16:3647594	VUS (PM2,PP3)	VUS (PM2 - Mo; PP3,BP1 - Su)	VUS	0.48	0.12	0.00004	0.00023
**P2242**	*SLX4*	AR	FA	NM_032444.4:c.2484G>C (p.Glu828Asp)	16:3641155	VUS (PM2,PP3)	VUS (PM2 - Mo; PP3,BP1 - Su)	VUS	0.52	0.37	0.00013	0.00111
**P2252**	*SLX4*	AR	FA	NM_032444.4:c.1925-2_1925-1del splicing	16:3645694	VUS (PM2)	P (PVS1 - VS; PM2 - Mo; PP3 - Su)	LP	0.41	0	0.00001	0.00009
**2166**	*TERT**	AD/AR	DKC, myeloid malignancies	NM_198253.3:c.604G>A (p.Ala202Thr)	5:1294397	VUS (PM2)	VUS (PM1,PM2 - Mo; PP5; BP4 - Su)	VUS	0.57	-	0.00033	0
**P2215**	*TP53*	AD	Li-Fraumeni syndrome	NM_000546.6:c.704A>G (p.Asn235Ser)	17:7577577	VUS (PM1,PM2,PP3,BP6)	VUS (PM1,PM5 - Mo; PP2, PP5 - Su; BS1,BS2 - St; BP4 - Su; BP6 - VS)	VUS	0.38	0.08	0.00018	0.00028
**P2224**	*TP53*	AD	Li-Fraumeni syndrome	NM_000546.6:c.733G>A (p.Gly245Ser)	17:7577548	LP (PM1,PM2,PM5,PP3, PP5)	P (PM1 - St; PM2,PM5 - Mo: PP2,PP3 - Su; PP5 - VS)	P	0.42	0.22	0	0
**2181**	*UBE2T*	AR	FA	NM_014176.4:c.106G>A (p.Ala36Thr)	1:202304777	LP (PM1,PM2,PP2,PP3)	VUS (PM1,PM2 - Mo; PP3 - Su)	VUS	0.56	-	0.00019	0.00018
**P2230**	*UBE2T*	AR	FA	NM_014176.4:c.444G>C (p.Glu148Asp)	1:202302162	VUS (PM2,PP2,PP3)	VUS (PM1,PM2 - Mo; BP4 - Su)	VUS	0.59	0.01	0.00008	0.00028

Supplementary Table S5. Variants in patients with >50% blasts. Found variants in pediatric patients with >50% blasts in the blood sample interpreted as germline based on the available knowledge. LP, likely pathogenic; P, pathogenic; VAF, variant allele frequency; VUS, variant of uncertain significance.

Patient	Blasts in sample	VAF	Bone marrow karyotype	Gene	Position (GRCh37)	Variant	ACMG Intervar	ACMG Varsome	Conclusion of pathogenicity	MAF gnomAD all	MAF gnomAD Finns	cBioPortal somatic	ClinVar germline submissions	Notes
**P2209**	0.78	0.47	46,XY,inv(1),i(8)	*CHEK2*	22:29091856	NM_007194.4:c.1100del (p.Thr367MetfsTer15)	VUS	P	P	0.00205	0.00874	No	58	Known germline variant enriched in Finns [66,67]
**P2218**	0.96	0.43	46,XX	*FANCM*	14:45658326	NM_020937.4:c.5101C>T (p.Gln1701Ter)	VUS	P	P	0.00129	0.00823	No	8	Known germline variant enriched in Finns, breast cancer risk. [68]
**P2220**	0.97	0.48	N/A	*BRIP1*	17:59760966	NM_032043.3:c.3440dup (p.Asn1147LysfsTer2)	VUS	P	LP	0.00009	0.00068	No	4	Only found as germline previously
**P2223**	0.83	0.33	46,XY,t(9;22)(q34; q11),-4.-21,+2mar	*SBDS*	7:66459197	NM_016038.4:c.258+2T>C	P	P	P	0.00397	0.00958	No	40	Known germline variant enriched in Finns
**P2234**	0.96	0.44	46,XY,t(4;11)	*DNAJC21*	5:34949702	NM_194283.4:c.1240G>T (p.Gly414Ter)	VUS	LP	LP	0.00041	0.00355	No	1	Only found as germline previously, variant enriched in Finns
		0.45		*SBDS*	7:66459197	NM_016038.4:c.258+2T>C	P	P	P	0.00397	0.00958	No	40	Known germline variant enriched in Finns
**P2235**	0.71	0.41	46,XX	*BLM*	15:91312417	NM_000057.4:c.2362C>A (p.Leu788Ile)	VUS	VUS	VUS	0.00093	0.00587	No	6	Variant enriched in Finns
**P2253**	0.55	0.76	60,XY,+X,+X,+Y,+Y, +1,+4,+10,+21,+21, +21+21,(inc)(6/30) / 52,XY,+X,+Y,+1, +10,-14,+21,+21, +mar(12/30) / 26,XY,-1,-2, -3,-4, -5,-6,-7,-8,-9,-11, -12,-13,-14,-15,-16, -17,-18,-19,-20, -22 (8/30)	*FANCM*	14:45644296	NM_020937.4:c.2339A>C (p.Glu780Ala)	VUS	VUS	VUS	0.00003	0	No	1	Only found as germline previously. same allele frequency in both variants correlating with loss of chr14 in some clones
		0.76		*FANCM*	14:45644346	NM_020937.4:c.2389C>G (p.Pro797Ala)	VUS	VUS	VUS	0.00003	0	No	1	Only found as germline previously, also the same frequency in gnomAD for both variants, suggesting linkage
**P2257**	0.87	0.42	45-47,XX,+mar	*CHEK2*	22:29091856	NM_007194.4:c.1100del (p.Thr367MetfsTer15)	VUS	P	P	0.00205	0.00874	No	58	Known germline variant enriched in Finns [66,67]

Supplementary Table S6. Enrichment of P/LP variants. Enrichment of pathogenic and likely pathogenic variants in the ALL patients compared to gnomAD Finns (non-cancer). *p<0.05, **p<0.01, ***p<0.001. OR, odds ratio; CI, confidence interval; q, Benjamini-Hochberg adjusted p-value.

Patient groups and gene panels	ALL variants	GnomAD variants	p-value	q	CI 95%	OR
adults hematological malignancy genes	3	361	0.45940	0.45940	0.3-4.64	1.50
adults hematological malignancy + cancer genes	8	587	**0.01741***	0.031338	1.07-5.6	2.63
adults cancer genes	5	226	**0.00937****	0.021082	1.3-10.5	4.18
pediatric patients hematological malignancy genes	7	361	**0.03051***	0.044511	0.96-5.37	2.47
pediatric patients hematological malignancy + cancer genes	11	587	**0.00777****	0.021083	1.20-4.80	2.52
pediatric patients cancer genes	4	226	0.1116	0.125550	0.59-6.08	2.25
all patients hematological malignancy genes	10	361	**0.03462***	0.044511	0.97-4.02	2.09
all patients hematological malignancy + cancer genes	19	587	**0.00071*****	0.006390	1.49-4.21	2.56
all patients cancer genes	9	226	**0.00456****	0.020520	1.34-6.03	3.03

Supplementary Table S7. Enrichment of P/LP variants in each gene. Enrichment of pathogenic or likely pathogenic variants in each gene in ALL patients compared to general population (gnomad Finns non-cancer). *p<0.05, **p<0.01; OR, odds ratio; CI, confidence interval; trunc, protein-truncating variants; q, Benjamini-Hochberg adjusted p-value.

**Gene**	**Age group**	**p-value**	**q**	**OR**	**CI 95%**
TP53	Adult (n=0)				
TP53	Pediatric (n=1)	0.05454	0.1308960	20.82	0.45-173.21
TP53	Total (n=1)	0.09076	0.1815200	12.21	0.26-101.21
SDHC	Adult (n=0)				
SDHC	Pediatric (n=1)	**0.015896***	0.0715320	124.67	1.58-8880.31
SDHC	Total (n=1)	**0.026816***	0.0877615	73.22	0.93-5435.24
RUNX1	Adult (n=1)	**0.005608****	0.0511128	Inf	4.55-
RUNX1	Pediatric (n=0)				
RUNX1	Total (n=1)	**0.013499***	0.0694234	Inf	1.87-
PMS2	Adult (n=1)	**0.022246***	0.0800856	59.44	1.13-733.19
PMS2	Pediatric (n=1)	**0.03154***	0.0946200	41.61	0.79-526.27
PMS2	Total (n=2)	**0.001768****	0.0318240	48.91	4.08-434.55
MUTYH	Adult (n=2)	0.089195	0.1815200	4.13	0.49-15.69
MUTYH	Pediatric (n=4)	**0.006107****	0.0511128	5.83	1.54-15.72
MUTYH	Total (n=6)	**0.001641****	0.0318240	5.12	1.82-11.74
LZTR1	Adult (n=0)				
LZTR1	Pediatric (n=1)	0.356722	0.5837269	2.31	0.06-13.6
LZTR1	Total (n=1)	0.526884	0.75871230	1.35	0.03-7.93
FANCM	Adult (n=0)				
FANCM	Pediatric (n=1)	0.548387	0.7593051	1.27	0.03-7.34
FANCM	Total (n=1)	1	1	0.74	0.02-4.28
ERCC6L2 trunc	Adult (n=2)	0.140063	0.2521134	3.12	0.37-11.77
ERCC6L2 trunc	Pediatric (n=1)	0.606155	0.8082067	1.08	0.03-6.23
ERCC6L2 trunc	Total (n=3)	0.213779	0.3664783	1.92	0.39-5.8
BRCA1	Adult (n=1)	**0.022246***	0.0800856	59.44	1.13-733.19
BRCA1	Pediatric (n=0)				
BRCA1	Total (n=1)	0.052915	0.1308960	24.43	0.46-304.74
SBDS	Adult (n=0)				
SBDS	Pediatric (n=4)	0.090208	0.1815200	2.42	0.65-6.41
SBDS	Total (n=4)	0.376726	0.5896581	1.41	0.38-3.71
DNAJC21 trunc	Adult (n=1)	0.404582	0.6068730	1.96	0.05-11.38
DNAJC21 trunc	Pediatric (n=4)	**0.007099****	0.0511128	5.57	1.47-15.01
DNAJC21 trunc	Total (n=5)	**0.009763****	0.0585780	4.07	1.28-9.95
CHEK2 trunc	Adult (n=1)	1	1	0.79	0.02-4.55
CHEK2 trunc	Pediatric (n=5)	**0.036334***	0.1006172	2.84	0.9-6.86
CHEK2 trunc	Total (n=6)	0.134704	0.2521134	1.99	0.72-4.44

SUPPLEMENTARY REFERENCES

1. Wartiovaara-Kautto, U., Hirvonen, E. A. M., Pitkänen, E., Heckman, C., Saarela, J., Kettunen, K., *et al.* Germline alterations in a consecutive series of acute myeloid leukemia. *Leukemia* **32**, 2282–2285 (2018).

2. Li, H. & Durbin, R. Fast and accurate short read alignment with Burrows-Wheeler transform. *Bioinformatics* **25**, 1754–1760 (2009).

3. McKenna, A., Hanna, M., Banks, E., Sivachenko, A., Cibulskis, K., Kernytsky, A., *et al.* The genome analysis toolkit: A MapReduce framework for analyzing next-generation DNA sequencing data. *Genome Res.* **20**, 1297–1303 (2010).

4. Furutani, E. & Shimamura, A. Genetic predisposition to MDS: Diagnosis and management. *Hematol. (United States)* **2019**, 110–119 (2019).

5. Speedy, H. E., Kinnersley, B., Chubb, D., Broderick, P., Law, P. J., Litchfield, K., *et al.* Germ line mutations in shelterin complex genes are associated with familial chronic lymphocytic leukemia. *Blood* **128**, 2319–2326 (2016).

6. Galera, P., Dulau-Florea, A. & Calvo, K. R. Inherited thrombocytopenia and platelet disorders with germline predisposition to myeloid neoplasia. *International Journal of Laboratory Hematology* vol. 41 131–141 (2019).

7. Gumy-Pause, F., Wacker, P. & Sappino, A. P. ATM gene and lymphoid malignancies. *Leukemia* vol. 18 238–242 (2004).

8. Taylor, A. M. R., Metcalfe, J. A., Thick, J. & Mak, Y. F. Leukemia and lymphoma in ataxia telangiectasia. *Blood* vol. 87 423–438 (1996).

9. Schütte, P., Möricke, A., Zimmermann, M., Bleckmann, K., Reismüller, B., Attarbaschi, A., *et al.* Preexisting conditions in pediatric ALL patients: Spectrum, frequency and clinical impact. *Eur. J. Med. Genet.* **59**, 143–151 (2016).

10. Pui, C. H., Nichols, K. E. & Yang, J. J. Somatic and germline genomics in paediatric acute lymphoblastic leukaemia. *Nat. Rev. Clin. Oncol.* **16**, 227–240 (2019).

11. Adams, M. Acute Myeloid Leukaemia after Treatment for Acute Lymphoblastic Leukaemia in Girl with Bloom Syndrome. *J. Genet. Syndr. Gene Ther.* **04**, (2013).

12. Alter, B. P. Fanconi anemia and the development of leukemia. *Best Practice and Research: Clinical Haematology* vol. 27 214–221 (2014).

13. Nalepa, G. & Clapp, D. W. Fanconi anaemia and cancer: An intricate relationship. *Nature Reviews Cancer* vol. 18 168–185 (2018).

14. Kuchenbaecker, K. B., Hopper, J. L., Barnes, D. R., Phillips, K. A., Mooij, T. M., Roos-Blom, M. J., *et al.* Risks of breast, ovarian, and contralateral breast cancer for BRCA1 and BRCA2 mutation carriers. *JAMA - J. Am. Med. Assoc.* **317**, 2402–2416 (2017).

15. Pouliot, G. P., Degar, J., Hinze, L., Kochupurakkal, B., Vo, C. D., Burns, M. A., *et al.* Fanconi-BRCA pathway mutations in childhood T-cell acute lymphoblastic leukemia. *PLoS One* **14**, (2019).

16. Tawana, K., Rio-Machin, A., Preudhomme, C. & Fitzgibbon, J. Familial CEBPA-mutated acute myeloid leukemia. *Seminars in Hematology* vol. 54 87–93 (2017).

17. Lewinsohn, M., Brown, A. L., Weinel, L. M., Phung, C., Rafidi, G., Lee, M. K., *et al.* Novel germ line DDX41 mutations define families with a lower age of MDS/AML onset and lymphoid malignancies. *Blood* **127**, 1017–1023 (2016).

18. Heiss, N. S., Knight, S. W., Vulliamy, T. J., Klauck, S. M., Wiemann, S., Mason, P. J., *et al.* X-linked dyskeratosis congenita is caused by mutations in a highly conserved gene with putative nucleolar functions. *Nat. Genet.* **19**, 32–38 (1998).

19. Bezzerri, V. & Cipolli, M. Shwachman-Diamond Syndrome: Molecular Mechanisms and Current Perspectives. *Molecular Diagnosis and Therapy* vol. 23 281–290 (2019).

20. Tummala, H., Kirwan, M., Walne, A. J., Hossain, U., Jackson, N., Pondarre, C., *et al.* ERCC6L2 mutations link a distinct bone-marrow-failure syndrome to DNA repair and mitochondrial function. *Am. J. Hum. Genet.* **94**, 246–256 (2014).

21. Douglas, S. P. M., Siipola, P., Kovanen, P. E., Pyörälä, M., Kakko, S., Savolainen, E.-R., *et al.* ERCC6L2 defines a novel entity within inherited acute myeloid leukemia. *Blood* **133**, 2724–2728 (2019).

22. Moriyama, T., Metzger, M. L., Wu, G., Nishii, R., Qian, M., Devidas, M., *et al.* Germline genetic variation in ETV6 and risk of childhood acute lymphoblastic leukaemia: A systematic genetic study. *Lancet Oncol.* **16**, 1659–1666 (2015).

23. Di Paola, J. & Porter, C. C. ETV6-related thrombocytopenia and leukemia predisposition. *Blood* **134**, 663–667 (2019).

24. Topka, S., Vijai, J., Walsh, M. F., Jacobs, L., Maria, A., Villano, D., *et al.* Germline ETV6 Mutations Confer Susceptibility to Acute Lymphoblastic Leukemia and Thrombocytopenia. *PLoS Genet.* **11**, 1–14 (2015).

25. Zhang, M. Y., Churpek, J. E., Keel, S. B., Walsh, T., Lee, M. K., Loeb, K. R., *et al.* Germline ETV6 mutations in familial thrombocytopenia and hematologic malignancy. *Nat. Genet.* **47**, 180–185 (2015).

26. Noetzli, L., Lo, R. W., Lee-Sherick, A. B., Callaghan, M., Noris, P., Savoia, A., *et al.* Germline mutations in ETV6 are associated with thrombocytopenia, red cell macrocytosis and predisposition to lymphoblastic leukemia. *Nat. Genet.* **47**, 535–538 (2015).

27. de Smith, A. J., Lavoie, G., Walsh, K. M., Aujla, S., Evans, E., Hansen, H. M., *et al.* Predisposing germline mutations in high hyperdiploid acute lymphoblastic leukemia in children. *Genes, Chromosom. Cancer* **58**, 723–730 (2019).

28. Costa, L. Da, Leblanc, T. & Mohandas, N. Diamond-Blackfan anemia. *Blood* vol. 136 1262–1273 (2020).

29. McReynolds, L. J., Calvo, K. R. & Holland, S. M. Germline GATA2 Mutation and Bone Marrow Failure. *Hematology/Oncology Clinics of North America* vol. 32 713–728 (2018).

30. Churchman, M. L., Qian, M., te Kronnie, G., Zhang, R., Yang, W., Zhang, H., *et al.* Germline Genetic IKZF1 Variation and Predisposition to Childhood Acute Lymphoblastic Leukemia. *Cancer Cell* **33**, 937-948.e8 (2018).

31. Papaemmanuil, E., Hosking, F. J., Vijayakrishnan, J., Price, A., Olver, B., Sheridan, E., *et al.* Loci on 7p12.2, 10q21.2 and 14q11.2 are associated with risk of childhood acute lymphoblastic leukemia. *Nat. Genet.* **41**, 1006–1010 (2009).

32. de Smith, A. J., Walsh, K. M., Morimoto, L. M., Francis, S. S., Hansen, H. M., Jeon, S., *et al.* Heritable variation at the chromosome 21 gene ERG is associated with acute lymphoblastic leukemia risk in children with and without Down syndrome. *Leukemia* vol. 33 2746–2751 (2019).

33. Trevĩo, L. R., Yang, W., French, D., Hunger, S. P., Carroll, W. L., Devidas, M., *et al.* Germline genomic variants associated with childhood acute lymphoblastic leukemia. *Nat. Genet.* **41**, 1001–1005 (2009).

34. Wiemels, J. L., Walsh, K. M., de Smith, A. J., Metayer, C., Gonseth, S., Hansen, H. M., *et al.* GWAS in childhood acute lymphoblastic leukemia reveals novel genetic associations at chromosomes 17q12 and 8q24.21. *Nat. Commun.* **9**, 286 (2018).

35. Altmann, T. & Gennery, A. R. DNA ligase IV syndrome; a review. *Orphanet Journal of Rare Diseases* vol. 11 1–7 (2016).

36. Bluteau, D., Masliah-Planchon, J., Clairmont, C., Rousseau, A., Ceccaldi, R., D'Enghien, C. D., *et al.* Biallelic inactivation of REV7 is associated with Fanconi anemia. *J. Clin. Invest.* **126**, 3580–3584 (2016).

37. Sanders, M. A., Chew, E., Flensburg, C., Zeilemaker, A., Miller, S. E., Al Hinai, A. S., *et al.* MBD4 guards against methylation damage and germ line deficiency predisposes to clonal hematopoiesis and early-onset AML. *Blood* **132**, 1526–1534 (2018).

38. Ripperger, T., Beger, C., Rahner, N., Sykora, K. W., Bockmeyer, C. L., Lehmann, U., *et al.* Constitutional mismatch repair deficiency and childhood leukemia/lymphoma - report on a novel biallelic MSH6 mutation. *Haematologica* **95**, 841–844 (2010).

39. Asensio-Juárez, G., Llorente-González, C. & Vicente-Manzanares, M. Linking the Landscape of MYH9-Related Diseases to the Molecular Mechanisms that Control Non-Muscle Myosin II-A Function in Cells. *Cells* vol. 9 (2020).

40. Pasic, S., Vujic, D., Fiorini, M. & Notarangelo, L. T-cell lymphoblastic leukemia/lymphoma in Nijmegen breakage syndrome. *Haematologica* **89**, (2004).

41. Naseem, S., Varma, N., Marwaha, R. & Agarwal, P. T-lineage acute lymphoblastic leukemia and parvovirus infection in a child with neurofibromastosis-1. *Indian J. Pathol. Microbiol.* **56**, 446 (2013).

42. Galbiati, M., Lettieri, A., Micalizzi, C., Songia, S., Morerio, C., Biondi, A., *et al.* Natural history of acute lymphoblastic leukemia in neurofibromatosis type 1 monozygotic twins. *Leukemia* vol. 27 1778–1781 (2013).

43. Nachmani, D., Bothmer, A. H., Grisendi, S., Mele, A., Bothmer, D., Lee, J. D., *et al.* Germline NPM1 mutations lead to altered rRNA 2′-O-methylation and cause dyskeratosis congenita. *Nat. Genet.* **51**, 1518–1529 (2019).

44. Shah, S., Schrader, K. A., Waanders, E., Timms, A. E., Vijai, J., Miething, C., *et al.* A recurrent germline PAX5 mutation confers susceptibility to pre-B cell acute lymphoblastic leukemia. *Nat. Genet.* **45**, 1226–1231 (2013).

45. Auer, F., Rüschendorf, F., Gombert, M., Husemann, P., Ginzel, S., Izraeli, S., *et al.* Inherited susceptibility to pre B-ALL caused by germline transmission of PAX5 c.547G>A. *Leukemia* **28**, 1136–1138 (2014).

46. Cavé, H., Caye, A., Strullu, M., Aladjidi, N., Vignal, C., Ferster, A., *et al.* Acute lymphoblastic leukemia in the context of RASopathies. *Eur. J. Med. Genet.* **59**, 173–178 (2016).

47. Kratz, C. P., Rapisuwon, S., Reed, H., Hasle, H. & Rosenberg, P. S. Cancer in Noonan, Costello, cardiofaciocutaneous and LEOPARD syndromes. *Am. J. Med. Genet. Part C Semin. Med. Genet.* **157**, 83–89 (2011).

48. Marconi, C., DI Buduo, C. A., LeVine, K., Barozzi, S., Faleschini, M., Bozzi, V., *et al.* Loss-of-function mutations in PTPRJ cause a new form of inherited thrombocytopenia. *Blood* **133**, 1346–1357 (2019).

49. Siitonen, A. H., Sotkasiira, J., Biervliet, M., Benmansour, A., Capri, Y., Cormier-Daire, V., *et al.* The mutation spectrum in RECQL4 diseases. *Eur. J. Hum. Genet.* **17**, 151–158 (2009).

50. Knies, K., Inano, S., Ramírez, M. J., Ishiai, M., Surrallés, J., Takata, M., *et al.* Biallelic mutations in the ubiquitin ligase RFWD3 cause Fanconi anemia. *J. Clin. Invest.* **127**, 3013–3027 (2017).

51. Mirabello, L., Khincha, P. P., Ellis, S. R., Giri, N., Brodie, S., Chandrasekharappa, S. C., *et al.* Novel and known ribosomal causes of Diamond-Blackfan anaemia identified through comprehensive genomic characterisation. *J. Med. Genet.* **54**, 417–425 (2017).

52. Ikeda, F., Yoshida, K., Toki, T., Uechi, T., Ishida, S., Nakajima, Y., *et al.* Exome sequencing identified RPS15A as a novel causative gene for Diamond-Blackfan anemia. *Haematologica* vol. 102 e93–e96 (2017).

53. Jahan, D., Hasan, M. M. Al & Haque, M. Diamond-Blackfan anemia with mutation in RPS19: A case report and an overview of published pieces of literature. *Journal of Pharmacy and Bioallied Sciences* vol. 12 163–170 (2020).

54. Linden, T., Schnittger, S., Groll, A. H., Juergens, H. & Rossig, C. Childhood B-cell precursor acute lymphoblastic leukaemia in a patient with familial thrombocytopenia and RUNX1 mutation. *Br. J. Haematol.* **151**, 528–530 (2010).

55. Prebet, T., Carbuccia, N., Raslova, H., Favier, R., Rey, J., Arnoulet, C., *et al.* Concomitant germ-line *RUNX1* and acquired *ASXL1* mutations in a T-cell acute lymphoblastic leukemia. *Eur. J. Haematol.* **91**, 277–279 (2013).

56. Narumi, S., Amano, N., Ishii, T., Katsumata, N., Muroya, K., Adachi, M., *et al.* SAMD9 mutations cause a novel multisystem disorder, MIRAGE syndrome, and are associated with loss of chromosome 7. *Nat. Genet.* **48**, 792–797 (2016).

57. Davidsson, J., Puschmann, A., Tedgård, U., Bryder, D., Nilsson, L. & Cammenga, J. SAMD9 and SAMD9L in inherited predisposition to ataxia, pancytopenia, and myeloid malignancies. *Leukemia* vol. 32 1106–1115 (2018).

58. Cheah, J. J. C., Brown, A. L., Schreiber, A. W., Feng, J., Babic, M., Moore, S., *et al.* A novel germline SAMD9L mutation in a family with ataxia-pancytopenia syndrome and pediatric acute lymphoblastic leukemia. *Haematologica* **104**, e318–e321 (2019).

59. Nelson, A. S. & Myers, K. C. Diagnosis, Treatment, and Molecular Pathology of Shwachman-Diamond Syndrome. *Hematology/Oncology Clinics of North America* vol. 32 687–700 (2018).

60. Perez-Garcia, A., Ambesi-Impiombato, A., Hadler, M., Rigo, I., LeDuc, C. A., Kelly, K., *et al.* Genetic loss of SH2B3 in acute lymphoblastic leukemia. *Blood* **122**, 2425–32 (2013).

61. Kirwan, M., Walne, A. J., Plagnol, V., Velangi, M., Ho, A., Hossain, U., *et al.* Exome sequencing identifies autosomal-dominant SRP72 mutations associated with familial aplasia and myelodysplasia. *Am. J. Hum. Genet.* **90**, 888–892 (2012).

62. Duployez, N., Goursaud, L., Fenwarth, L., Bories, C., Marceau-Renaut, A., Boyer, T., *et al.* Familial myeloid malignancies with germline TET2 mutation. *Leukemia* **34**, 1450–1453 (2020).

63. Powell, B. C., Jiang, L., Muzny, D. M., Treviño, L. R., Dreyer, Z. E., Strong, L. C., *et al.* Identification of TP53 as an acute lymphocytic leukemia susceptibility gene through exome sequencing. *Pediatr. Blood Cancer* **60**, E1 (2013).

64. Waanders, E., Scheijen, B., Jongmans, M. C. J., Venselaar, H., Van Reijmersdal, S. V., Van Dijk, A. H. A., *et al.* Germline activating TYK2 mutations in pediatric patients with two primary acute lymphoblastic leukemia occurrences. *Leukemia* **31**, 821–828 (2017).

65. Sullivan, K. E., Mullen, C. A., Blaese, R. M. & Winkelstein, J. A. A multiinstitutional survey of the Wiskott-Aldrich syndrome. *J. Pediatr.* **125**, 876–885 (1994).

66. Naslund-Koch, C., Nordestgaard, B. G. & Bojesen, S. E. Increased risk for other cancers in addition to breast cancer for CHEK2∗1100delC heterozygotes estimated from the copenhagen general population study. *J. Clin. Oncol.* **34**, 1208–1216 (2016).

67. Schmidt, M. K., Hogervorst, F., Van Hien, R., Cornelissen, S., Broeks, A., Adank, M. A., *et al.* Age-And tumor subtype-specific breast cancer risk estimates for CHEK2∗1100delC Carriers. *J. Clin. Oncol.* **34**, 2750–2760 (2016).

68. Kiiski, J. I., Tervasmäki, A., Pelttari, L. M., Khan, S., Mantere, T., Pylkäs, K., *et al.* FANCM mutation c.5791C>T is a risk factor for triple-negative breast cancer in the Finnish population. *Breast Cancer Res. Treat.* **166**, 217–226 (2017).
